# A Validation Study of a Smartphone-Based Finger Tapping Application for Quantitative Assessment of Bradykinesia in Parkinson’s Disease

**DOI:** 10.1371/journal.pone.0158852

**Published:** 2016-07-28

**Authors:** Chae Young Lee, Seong Jun Kang, Sang-Kyoon Hong, Hyeo-Il Ma, Unjoo Lee, Yun Joong Kim

**Affiliations:** 1 Department of Neurology, Hallym University Sacred Heart hospital, Hallym University College of Medicine, Hallym University, Anyang, Korea; 2 Department of Electronic Engineering, Hallym University, Chuncheon, Korea; 3 Hallym Institute of Translational Genomics & Bioinformatics, Hallym University Medical Center, Anyang, Korea; 4 ILSONG Institute of Life Science, Hallym University, Anyang, Korea; Duke University, UNITED STATES

## Abstract

**Background:**

Most studies of smartphone-based assessments of motor symptoms in Parkinson’s disease (PD) focused on gait, tremor or speech. Studies evaluating bradykinesia using wearable sensors are limited by a small cohort size and study design. We developed an application named smartphone tapper (SmT) to determine its applicability for clinical purposes and compared SmT parameters to current standard methods in a larger cohort.

**Methods:**

A total of 57 PD patients and 87 controls examined with motor UPDRS underwent timed tapping tests (TT) using SmT and mechanical tappers (MeT) according to CAPSIT-PD. Subjects were asked to alternately tap each side of two rectangles with an index finger at maximum speed for ten seconds. Kinematic measurements were compared between the two groups.

**Results:**

The mean number of correct tapping (MCoT), mean total distance of finger movement (T-Dist), mean inter-tap distance, and mean inter-tap dwelling time (IT-DwT) were significantly different between PD patients and controls. MCoT, as assessed using SmT, significantly correlated with motor UPDRS scores, bradykinesia subscores and MCoT using MeT. Multivariate analysis using the SmT parameters, such as T-Dist or IT-DwT, as predictive variables and age and gender as covariates demonstrated that PD patients were discriminated from controls. ROC curve analysis of a regression model demonstrated that the AUC for T-Dist was 0.92 (95% CI 0.88–0.96).

**Conclusion:**

Our results suggest that a smartphone tapping application is comparable to conventional methods for the assessment of motor dysfunction in PD and may be useful in clinical practice.

## Introduction

Bradykinesia is a core feature that is essential in the clinical diagnosis of Parkinson’s disease (PD). The motor section of the Unified Parkinson’s Disease Rating Scale (motor UPDRS) and its subscores are the current gold standard for the assessment of motor dysfunction in PD. Motor UPDRS is an established method, but inter- and intra-rater reliability are only acceptable in well-trained persons. The timed tapping test using mechanical tappers in the Core assessment program for surgical interventional therapies in Parkinson's disease (CAPSIT-PD) protocol [1] is an objective method for the evaluation of bradykinesia, but no mechanical tapper instrument is widely used in routine clinical practice. Methods to evaluate tremor, bradykinesia, voice change, postural instability, and gait disturbance in PD were introduced with the development of sensor technologies using the accelerometer in custom-grade smartphone or wearable sensors (S1 Table) [2–22]. Most of these studies assessed gait disturbance or tremor, and few studies performed smartphone-based assessments of bradykinesia [2–11]. Studies using wearable sensors to assess bradykinesia have the following limitations: study design with a PD group only; the primary aim focused on technical points of view rather than clinical applicability; and a small number of study participants with factors that affected tapping speed were neglected.

We developed a smartphone application for a timed finger-tapping test (SmT) to determine the applicability of SmT for clinical purposes. SmT parameters were compared between PD cases and controls, and correlation analyses of parameters with current gold standard methods were performed. We also explored whether PD could be discriminated from controls using SmT parameters.

## Subjects and Methods

### Subjects

The Institutional Review Board of the Hallym University Sacred Heart Hospital approved this study. Participants were signed on a written informed consent which was approved by the same IRB. A total of 87 controls and 57 patients with PD were recruited. PD was diagnosed using the criteria based on the UK brain bank [23]. Healthy controls lacked Parkinsonism, thyroid disease, cerebrovascular diseases, or essential tremor on neurological examinations and history taking. Patients with cognitive impairment were excluded. All patients except for 6 drug-naïve patients were tested under medication. A total of 10 patients were reported to have ON-OFF stages. All of these patients were tested in an ON stage. Demographic information, including handedness, were collected for all of the subjects. Handedness was determined using the Edinburgh handedness inventory [24]. Clinical information for the PD group, including motor severity as evaluated using the motor UPDRS, was collected according to the standardized protocol. Table 1 summarizes the demographic data of the PD and control groups.

**Table 1:** Demographic profile of patients with idiopathic Parkinson’s disease and controls.

	PD	Controls	P values
Number of subjects, n	57	87	-
Age-at-study, years (SD)	65.4 (9.0)	53.4 (14.8)	<0.01
Gender, male %	59.6	39.1	<0.05
Handedness, right/left/bilateral	53/0/4	84/3/0	<0.01
PD duration, months (SD)	58.0 (45.1)	-	-
Medication status		-	-
levodopa naïve, n	6	-	-
On dopaminergic medication, n	51	-	-
LEDD ranges, mg	100–1600	-	-
LEDD mean (SD), mg	622.09 (365.71)	-	-
Hoehn & Yahr stages (1/2/2.5/3)	3/24/16/14	-	-
Motor UPDRS scores, mean	20.0 (10.6)	-	-

PD, idiopathic Parkinson’s disease, LEDD, levodopa equivalent dose (Tomlinson, C.L., et al., Mov Disord, 2010. 25(15): p. 2649–53.), motor UPDRS, unified Parkinson’s disease rating scale.

### Timed tapping test using mechanical tappers

Timed tapping tests using a mechanical tapper were performed according to the CAPSIT-PD protocol [1]. Briefly, two mechanical finger tappers (WW-1597-NP, PAR Inc. Lutz, USA) were fixed on 250 X 450 square millimeter board separated by 300 millimeters. Tapping on one tapper (MeT1) was performed for 10 seconds, and the alterative tapping of two tappers (MeT2) was performed for 20 seconds. Each trial of MeT1 and MeT2 was repeated three times at maximal speed.

### Timed tapping test using a smartphone tapper

Two authors (S.J.K. and U.L.) developed a smartphone application for the timed tapping test and installed the application in LG Optimus G smartphones with Android OS. This application is available at the following link: https://sites.google.com/site/neurorehabict/downloads/ftapp. This application consists of two rectangles of 30 by 45 millimeters, separated by 15 millimeters (S1 Fig). Smartphone timed tapping test subjects were asked to alternately tap each side of the rectangles using an index finger at their fastest speed for ten seconds. The same tapping tasks were repeated three times for each hand. The results of the tests, including the number of trials, screen pixel position of each tap (X, Y coordinates), time of each tap, and the number of taps per trial, were transferred to Google drive via the internet. Kinematic measurements, such as inter-tap distance, inter-tap dwelling time (time lapse between two consecutive taps), total distance of a finger movement and tapping speed of each movement of a finger, were calculated from the recorded parameters. Briefly, inter-tap distance, which refers to the distance between two consecutive tap positions on the screen, was calculated using Pythagoras' theorem. The total distance of a finger movement was calculated indirectly by summation of all inter-tap distances in each tapping task. Tapping errors (tapping outside of squares) were determined using the position of each tap. Mean and median values of parameters in three trials were used to analyze the data.

### Statistical analyses

The means of all continuous variables between PD and control groups were compared using an independent t-test or Mann-Whitney U test. Analyses were conducted for the better hand, the worse hand and the average scores of both hands. The better hand and the worse hand were assigned based on the following rules. The dominant hand in controls was assigned as the better hand, and the non-dominant hand was the worse hand. The better or worse hand in PD patients was determined based on motor UPDRS score regardless of handedness. The hand with the higher mean number of taps was assigned as the better hand in cases with clinically symmetric motor signs. The asymmetry index of a parameter was calculated as follows: asymmetry index = 100 x (better-worse)/(better + worse). T-tests were used for comparisons of means between groups. Univariate analyses were conducted to determine the effects of sex, age, and hand dominance on the number of taps. Linear regression analysis was used to determine the relationship between the number of taps obtained using the smartphone tapper and motor UPDRS scores or the mean number of taps obtained using the mechanical tapper. Receiver operating (ROC) curve analyses were performed to investigate the utility of any of the above parameters in discriminating PD patients from controls. A p<0.05 was considered statistically significant in all tests except for a multiple comparison of parameters obtained from smartphone tappers or mechanical tappers between PD and controls (Table 2), where a Bonferroni corrected p value of <0.00139 was considered significant. All statistical analyses were performed using R (version 3.2.4, Graduate School of Public Health, Seoul National University, Seoul, Korea, http://healthstat.snu.ac.kr/CRAN/).

**Table 2:** Comparison of parameters obtained from mechanical tappers and smartphone tappers between idiopathic Parkinson’s disease and controls.

Parameters (units)			hand	PD	Control	p value*
Mechanical tapper	No. of taps in one-point tapping		Average	19.5 (4.5)	28.0 (5.2)	**<0.0001**
			Better	20.7 (4.8)	29.4 (5.8)	**<0.0001**
			Worse	18.4 (4.5)	26.6 (4.9)	**<0.0001**
	No. of taps in two-points tapping		Average	47.8 (12.1)	55.8 (10.8)	**<0.0001**
			Better	49.5 (12.5)	58.4 (11.0)	**0.001**
			Worse	45.8 (12.4)	53.2 (11.7)	**<0.0001**
Smart-phone tapper	No. of correct taps	mean	Average	40.54 (7.92)	54.50 (11.53)	**<0.0001**
			Better	42.62 (8.98)	58.70 (13.26)	**<0.0001**
			Worse	38.46 (8.12)	50.29 (10.65)	**<0.0001**
		Variance in three trials	Better	11.61 (37.70)	17.89 (41.07)	0.356
			Worse	4.66 (6.03)	15.93 (33.75)	0.003
		AI	Mean	0.06 (0.07)	0.07 (0.07)	0.488
	No. of tap error	Total number of tap error in 3 trials	Both	2.02 (3.75)	2.99 (4.45)	0.176
			Better	4.51 (6.43)	5.87 (6.66)	0.504
			Worse	2.49 (3.38)	2.89 (3.50)	0.225
		Mean number of tap error in each trial	Average	0.75 (1.1)	1.0 (1.1)	0.176
			Better	0.67 (1.2)	1.0 (1.5)	0.504
			Worse	0.83 (1.1)	1.0 (1.2)	0.225
		Variance of tap error in 3 trials	Average	1.4 (3.4)	1.8 (3.5)	0.666
			Better	1.3 (4.5)	1.6 (3.9)	0.956
			Worse	1.4 (3.7)	1.3 (2.3)	0.457
	Total distance of finger movement, mean (mm)		Average	807.60 (193.72)	1330.63 (341.11)	**<0.0001**
			Better	837.77 (210.26)	1423.98 (378.89)	**<0.0001**
			Worse	777.42 (194.63)	1237.28 (319.64)	**<0.0001**
			AI	0.03 (0.07)	0.06 (0.06)	<0.05
	Inter-tap distance of correct taps, mean		Average	19.77 (2.43)	23.51 (3.06)	**<0.0001**
			Better	20.02 (3.06)	24.05 (3.65)	**<0.0001**
			Worse	19.53 (2.65)	22.97 (3.16)	**<0.0001**
			AI	-0.01 (0.08)	0.02 (0.06)	<0.05
	Inter-tap distance of tap errors, mean (mm)		Better	25.32 (14.51)	34.85 (17.40)	0.0030
			Worse	27.03 (15.66)	33.99 (18.64)	0.0459
	Dwelling time in correct tap, mean (seconds)		Average	0.25 (0.06)	0.19 (0.05)	**<0.0001**
			Better	0.24 (0.05)	0.17 (0.05)	**<0.0001**
			Worse	0.26 (0.06)	0.20 (0.05)	**<0.0001**
			AI	-0.05 (0.07)	-0.08 (0.06)	<0.05
	Dwelling time in tap errors, mean (seconds)		Better	0.33(0.07)	0.23 (0.08)	**<0.0001**
			Worse	0.33 (0.07)	0.26 (0.10)	**0.0001**

Values are means and standard deviation in parenthesis. PD, idiopathic Parkinson’s disease; Average, average of both hands; Better, the better hand; Worse, the worse hand; Both, sum of both hands. AI, asymmetry index.

* T-test or Mann-Whitney U test. A Bonferroni corrected p value <0.00139, which is marked in bold, was considered statistically significant.

## Results

Table 2 summarizes the comparisons of the parameter means from MeT and SmT. Analyses of the parameters from the correct taps within the rectangles revealed that the mean number of taps was smaller, the total distance of finger movement and the inter-tap distance were shorter, and the inter-tap dwelling time was longer in the PD patients compared to the controls (p<0.00139). On the other hand, the analyses of the parameters from the tap errors revealed that only the mean dwelling time was significantly different between the two groups (p≤0.0001). The asymmetry indices were not significantly different in all the parameters: number of correct tap, total distance of finger movements, inter-tap distance of correct tap and inter-tap dwelling time of correct tap.

The number of taps using SmT highly correlated with mechanical tappers in PD and control groups regardless of hand laterality (R^2^ from 0.19 to 0.57, S2 Table). Notably, the correlation of tapping numbers using SmT was higher with the 2-points tapping task compared to the 1-point tapping task in control mechanical tappers. However, the degree of correlation in the number of taps in the PD group was similar between SmT and both tasks using mechanical tappers (MeT1 and MeT2). Table 3 summarizes the results of linear regression between the median number of taps using a smartphone tapper or mechanical tappers and the score or subscores of motor UPDRS, which is the gold standard method for the evaluation of motor severity of PD. The median number of taps in three trials of SmT, MeT1 and MeT2 significantly correlated with motor UPDRS scores. The correlation of the median number of taps from SmT with bradykinesia was the highest of the motor UPDRS subscores, but there was no correlation with the tremor subscore. A sub-analysis to correlate between the motor UPDRS score of each side of the limbs and the median number of taps from the ipsilateral or contralateral hand was performed (S3 Table). The bradykinesia subscore of the right limbs correlated better with the median tap number of the right hand than that of the left hand. However, the degree of correlation between the bradykinesia subscore of the left limbs were similar in both sides. The unilateral tremor scores did not correlate with the ipsilateral or contralateral timed tapping test results. The unilateral tremor score also did not correlate with the total number of tap error or variance of numbers of taps on the ipsilateral side. The means of the number of taps exhibited similar results.

**Table 3:** Results of linear regression between number of taps in three different timed tapping tests using a mechanical or smartphone tapper and motor scores of Unified Parkinson’s disease rating scale or its subscores in idiopathic Parkinson’s disease.

		Average				Better hand				Worse hand			
		Estimate	SE	R^2^	p value	Estimate	SE	R^2^	p value	Estimate	SE	R^2^	p value
motor UPDRS scores	MeT1P	-0.52	0.14	0.21	0.0004	-0.57	0.14	0.24	0.0002	-0.45	0.15	0.15	0.0030
	MeT2P	-0.21	0.05	0.25	< 0.0001	-0.22	0.05	0.24	0.0001	-0.21	0.05	0.24	0.0001
	SmT	-0.37	0.09	0.25	< 0.0001	-0.43	0.10	0.27	< 0.0001	-0.29	0.10	0.14	0.0049
bradykinesia subscore of motor UPDRS	MeT1P	-1.05	0.24	0.26	< 0.0001	-1.17	0.24	0.31	< 0.0001	-0.93	0.25	0.20	0.0005
	MeT2P	-0.41	0.09	0.29	< 0.0001	-0.43	0.09	0.27	< 0.0001	-0.40	0.09	0.29	< 0.0001
	SmT	-0.75	0.15	0.32	< 0.0001	-0.88	0.16	0.36	< 0.0001	-0.58	0.17	0.17	0.0013
rigidity subscore of motor UPDRS	MeT1P	-1.17	0.62	0.06	0.0657	-1.19	0.65	0.06	0.0739	-0.85	0.65	0.03	0.1967
	MeT2P	-0.57	0.23	0.10	0.0152	-0.57	0.25	0.09	0.0243	-0.55	0.23	0.10	0.0175
	SmT	-0.71	0.41	0.05	0.0895	-0.90	0.45	0.07	0.0503	-0.42	0.44	0.02	0.3448
tremor subscore of motor UPDRS	MeT1P	0.62	0.80	0.01	0.4417	0.82	0.83	0.02	0.3275	0.62	0.82	0.01	0.4530
	MeT2P	-0.02	0.30	0.00	0.9423	-0.05	0.32	0.00	0.8712	-0.02	0.30	0.00	0.9493
	SmT	0.03	0.52	0.00	0.9544	0.24	0.58	0.00	0.6798	0.17	0.55	0.00	0.7629
postural instability and gait disturbance subscore of motor UPDRS	MeT1P	-2.05	0.57	0.19	0.0006	-2.27	0.58	0.22	0.0003	-1.95	0.59	0.17	0.0016
	MeT2P	-0.74	0.21	0.18	0.0010	-0.77	0.23	0.17	0.0016	-0.74	0.21	0.19	0.0008
	SmT	-1.18	0.38	0.15	0.0031	-1.32	0.42	0.15	0.0028	-1.11	0.41	0.12	0.0086

MeT1P, one-point tap measure of a mechanical tapper; MeT2P, two-points tap measure of a mechanical tapper; SmT, smartphone tapper.

We determined the factors that affected the number of smartphone taps. Males and younger control group subjects exhibited higher numbers of smartphone taps (S4 and S5 Tables). Univariate analysis in the PD group revealed that PD duration, Hoehn & Yahr stage, and motor UPDRS score negatively correlated with the number of smartphone taps. However, multivariate analysis demonstrated that only motor UPDRS scores correlated negatively with the number of smartphone taps (S6 Table). Multivariate analyses were performed using the diagnosis of PD or control as the response variable, parameters obtained from smartphone tappers as predictive variables, and age and sex as covariates to investigate whether parameters derived from smartphone tappers could discriminate PD patients from controls. The mean number of correct taps, variance of tapping number in the worse hand, total distance of finger movements, inter-tap distance of correct taps, inter-tap distance of tap errors in the better hand, and inter-tap dwelling time discriminated PD patients from controls (Table 4).

**Table 4:** Results of multivariate analyses to determine smartphone tapper parameters discriminating idiopathic Parkinson’s disease from controls. Age and gender were used as covariates in each analysis.

Parameters		laterality	Estimate	SE	p value
No. of correct taps	Mean	Average	-0.15	0.03	<0.0001
		Better	-0.13	0.03	<0.0001
		Worse	-0.13	0.03	<0.0001
	Variance in 3 trials	Better	-0.01	0.01	0.3100
		Worse	-0.06	0.02	0.0113
	Asymmetry index	mean	-0.68	2.77	0.8044
No. of tap errors	total number	Both	-0.06	0.03	0.0672
		Better	-0.05	0.05	0.3383
		Worse	-0.08	0.06	0.1779
	Variance in 3 trials	Both	-0.08	0.06	0.2152
		Better	-0.05	0.05	0.3383
		Worse	-0.03	0.07	0.6570
Total distance of finger movement, mean		Average	0.00	0.00	<0.0001
		Better	0.00	0.00	<0.0001
		Worse	0.00	0.00	<0.0001
		Asymmetry index	-5.71	2.94	0.5216
Inter-tap distance of correct taps, mean		Average	-0.02	0.00	<0.0001
		Better	-0.02	0.00	<0.0001
		Worse	-0.02	0.00	<0.0001
		Asymmetry index	-5.97	2.92	0.0412
Inter-tap distance of tap errors, mean		Better	0.00	0.00	0.0059
		Worse	0.00	0.00	0.2504
Dwelling time of correct taps, mean		Average	24.31	5.71	<0.0001
		Better	24.65	5.68	<0.0001
		Worse	19.21	4.74	0.0001
		Asymmetry index	6.10	3.29	0.0637
Dwelling time of tap errors, mean		Better	17.84	4.62	0.0001
		Worse	6.07	2.80	0.0304

Average, average of both hands; Better, the better hand; Worse, the worse hand; Both, sum of both hands

**Fig 1:**
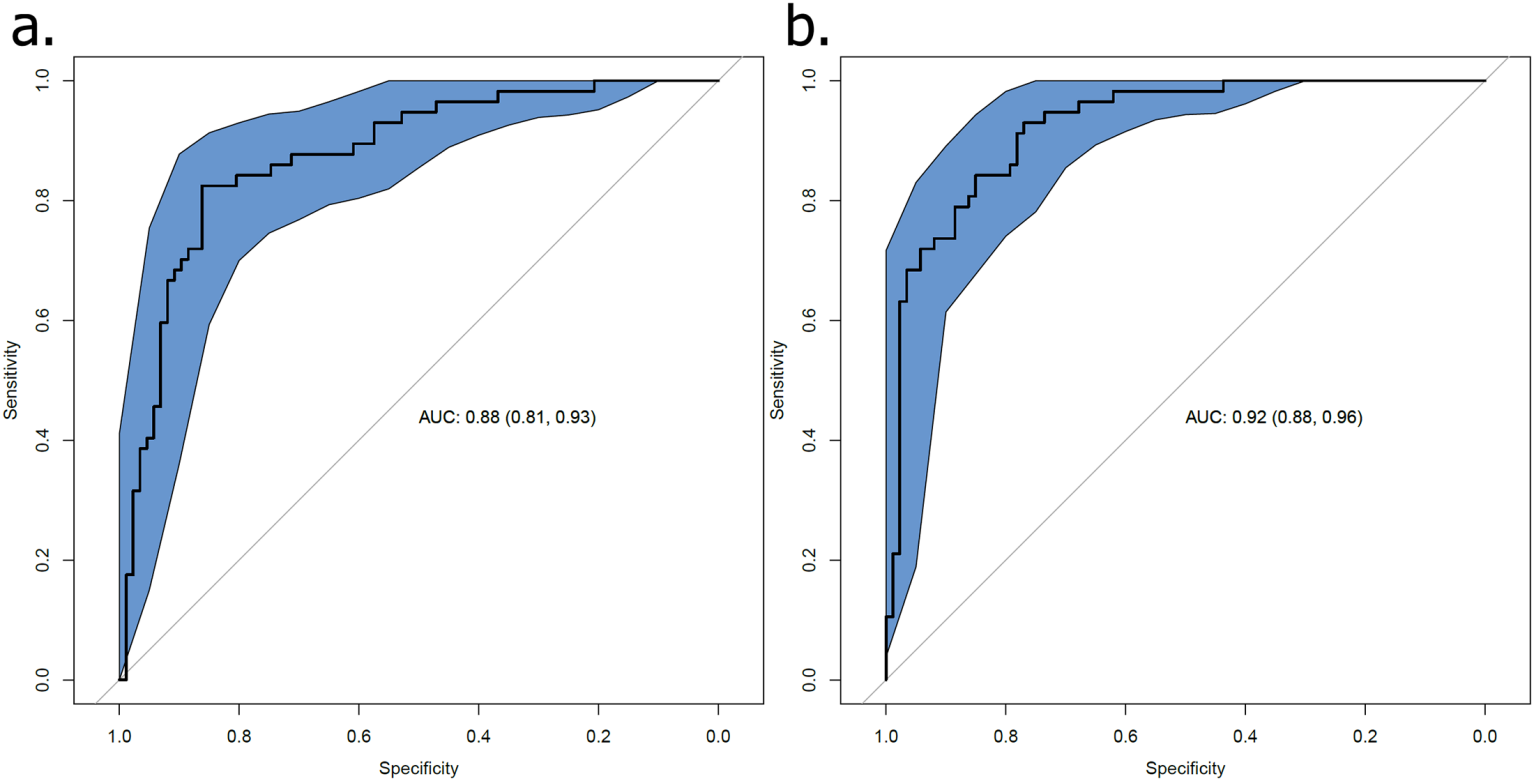
ROC curve analyses of multiple regression models to predict idiopathic Parkinson’s disease or control. Dwelling time (a) or total distance averaged for both sides (b) were predictive variables, and age and gender were used as covariates. Shaded areas represent 95% confidence intervals, which are indicated in parentheses.

We performed ROC curve analyses using a logistic regression model where diagnosis was set as a response variable, the mean of total distance or inter-tap dwelling time were predictive variables, and age and gender were covariates. ROC analyses using 2000 non-stratified bootstrap sampling demonstrated that the AUC for dwelling time was 0.88 (95% CI 0.82–0.93), and the AUC for the total distance of finger movements was 0.92 (95% CI 0.88–0.96) (Fig 1).

## Discussion

Our results demonstrated that the SmT parameters of slowness and smaller amplitude of finger tapping movements reflected the characteristics of bradykinesia of PD. The asymmetry indices related with the tapping speed and distance were not different between the PD and control groups because of our stringent p value threshold. The number of taps using SmT highly correlated with mUPDRS and its bradykinesia subscore. Several kinematic measurement devices using a smartphone application or a wearable sensor to capture characteristics of motor dysfunction in PD have been developed (S1 Table) [2–10, 12–21, 25]. Most kinematic studies using smartphones assessed gait disturbance or tremor rather than bradykinesia [3–7, 9, 10]. Wearable sensors or non-wearable devices are generally adopted for analyses of bradykinesia, and studies using smartphones are few [3, 9, 12, 14, 15, 21, 25, 26]. Studies using these devices suffer the following limitations for clinical applicability. The studies are also limited by their focus on the technical aspects of the device rather than the clinical application [10, 16, 19]. The number of participants is also small, and extensive analyses of kinematic parameters in controls, which are affected by age, sex and handedness, are lacking [3, 9, 12, 13, 16, 18, 19, 21, 22, 26]. Our study is the largest cohort of PD patients and controls to focus on the applicability of a smartphone application for the assessment of bradykinesia. Noyce et al. [25] reported a well-designed study to assess bradykinesia in PD patients using a computer keyboard, which reflected characteristic features of bradykinesia. Our study of SmT has advantages over this previous study because the total distance of finger movements and inter-tap distance, which reflect characteristic smaller amplitude movements in PD patients, were measured. Another important advantage of a smartphone application compared to a wearable sensor or computer keyboard is its easy availability, operability and mobility for patients and health care providers, which renders it more suitable for a large-scale field study.

Previous smartphone tapping tasks [3, 9] used an alternative tapping test using two fingers (index and middle finger) or a one-point finger tapping test using the index finger. In contrast, our finger tapping task with SmT was a two-points finger tapping test using one index finger. Notably, tapping numbers of SmT correlated better with the two-points tapping task using a mechanical tapper in controls despite the 20-fold difference in distance of the two tapping targets between SmT and mechanical tappers. However, we did not observe this different degree of correlation between the one-point and two-points tapping tests in PD, likely because of a floor effect. The correlation of tapping number using SmT with the bradykinesia subscore, but not the tremor subscore of mUPDRS supports the applicability of SmT in clinical practice. However, considering that the disease severity of our IPD patients was mild and moderate, a possible negative effect of tremor on an accurate measurement of the SmT tap number might have been under-estimated. Two small-scale pilot studies revealed the possibility of discriminating PD patients from controls using a machine-learning algorithm on smartphone-based measurements of motor function [3, 10]. We did not adopt a machine-learning algorithm, but we could explore whether SmT parameters could discriminate PD patients from controls in a larger data set of SmT parameters from cases and controls. The high AUC for total distance of finger movement in ROC curve analyses using a regression model suggests that SmT is an applicable screening test for PD. Additional parameters that are independent of the speed and distance of finger taps measured by SmT should be included. Alternatively, our SmT may provide additional parameters to the current smartphone-based gait or speech measurements to discriminate PD patients from controls. There are limitations in our study. Unfortunately, we failed to find parameters that reflected fatigue (decrement response) and hesitation (inter-tap irregularity), which are characteristics of motor dysfunction in PD. A recent pilot study by Arora analyzed hastening, faltering or freezing in the tapping patterns (arrythmokinesis) of a smartphone-based finger tapping tasks using two fingers, but the parameters of these analyses were not described in detail [3]. Total and median numbers of tap errors and variance, which we expected to reflect irregularity, were not different between two groups. Hesitation may have been incorporated in increased inter-tap dwelling time in SmT. Precise definitions of fatigue and hesitation in finger tapping tests in clinical settings may be needed to identify the parameters that reflect these parameters in SmT. Drug-naïve PD patients in early stages were only 10.5% of the population in our study, and differences of SmT parameters in de novo PD patients may be smaller than the differences in our data. Our study does not consider the possible effect of anti-parkinsonian medication on the tapping score, which may or may not be affected. Kraus et al (2005) demonstrated that plugging scores of a pegboard test, but not fast tapping scores, tracked changes that paralleled decrements in clinical scores. Plugging scores varied with the action of dopaminergic therapy, whereas a tapping score showed no therapeutic response [27]. Our study cannot be directly compared to this study because the SmT has several parameters other than the number of taps, and we did not include a pegboard test as a reference gold standard method. Whether SmT is applicable in determining levodopa responsiveness or monitoring the progression of motor dysfunction in PD should be explored in future studies. The attention function, which affects finger tapping speed, was not systemically evaluated or included in our analyses. The patient cohort was not well matched for age or gender, although both were adjusted in the multivariate analysis by including them as covariates.

In conclusion, our smartphone tapping application was comparable to conventional methods for the assessment of bradykinesia in PD, and this application may be useful in clinical practice or field studies.

## Supporting Information

S1 Fig The interface of a smartphone tapper used in this study. (TIF)

S1 Table Summary of smartphone application, non-wearable device and wearable sensors previously published in literatures. (DOCX)

S2 Table Results of linear regression between numbers of taps using two different methods of a mechanical tapper and smartphone tapper in idiopathic Parkinson’s disease and controls. (DOCX)

S3 Table Results of linear regression between number of taps in three different timed tapping tests using a mechanical or smartphone tapper and limb’s item sum of motor scores of Unified Parkinson’s disease rating scale in idiopathic Parkinson’s disease. (DOCX)

S4 Table Differences in number of taps in the smartphone tapper test according to sex in normal controls. (DOCX)

S5 Table Changes in number of taps in the smartphone tapper test according to age in normal controls. (DOCX)

S6 Table Factors affecting tap number in smartphone tapper test in patients with idiopathic Parkinson’s disease. (DOCX)
