# Supplementary material for: A Validation Study of a Smartphone-Based Finger Tapping Application for Quantitative Assessment of Bradykinesia in Parkinson’s Disease
Source: PLoS One. 2016 Jul 28;11(7):e0158852. doi: 10.1371/journal.pone.0158852 (PMC4965104; doi:10.1371/journal.pone.0158852)
Supplement: S1 Table — (DOCX) [file pone.0158852.s002.docx]

**S1 Table. Summary of smartphone application, non-wearable device and wearable sensors previously published in literatures.**

| Type of device | First author | Year | Type of sensor | Target motor symptom to assess | No. of parti-cipants (PD case : control) | References |
| --- | --- | --- | --- | --- | --- | --- |
| smartphone | Raknim P | 2016 | accelerometer | gait | 17:0 | Telemed J E Health. 2016;22:75-81 |
|  | Pan D | 2015 | accelerometer | gait, tremor | 40:0 | JMIR Mhealth Uhealth. 2015;3:e29 |
|  | Kostikis N | 2015 | accelerometer and gyroscope | tremor | 25:20 | IEEE J Biomed Health Inform. 2015;19:1835-42 |
|  | Ellis RJ | 2015 | accelerometer and gyroscope | gait | 12:12 | PLoS One. 2015;10:e0141694 |
|  | Arora S | 2015 | microphone, accelerometer | gait, tremor, speech, voice, posture, finger tapping, reaction time | 10:10 | Parkinsonism Relat Disord. 2015;21:650-3 |
|  | Printy BP | 2014 | accelerometer and gyroscope | bradykinesia | 26:0 | Conf Proc IEEE Eng Med Biol Soc. 2014;2014:2686-9 |
|  | Palmerini L | 2011 | accelerometer | gait (time up and go test) | 0:49 | Conf Proc IEEE Eng Med Biol Soc. 2011;2011:7179-82 |
| non-wearable device | Noyce AJ | 2014 | computer keyboard | bradykinesia | 58:93 | PLoS One. 2014;9:e96260 |
|  | Roy SH | 2011 | surface EMG and accelerometer | tremor, dyskinesia | 8:04 | Mov Disord. 2013;28:1080-7 |
|  | Taylor Tarvares AL | 2005 | musical keyboard | bradykinesia | 33:0 | Mov Disord. 2005;20:1286-98 |
| wearable sensor or device | Dai H | 2015 | accelerometer and gyroscope | bradykinesia | 8:7 | Biomed Eng Online. 2015;14:68 |
|  | Dai H | 2015 | accelerometer and gyroscope | tremor | 7:0 | Sensors (Basel). 2015;15:25055-71 |
|  | Roy SH | 2013 | surface EMG and accelerometer | tremor, dyskinesia | 8:4 | Mov Disord. 2013;28:1080-7 |
|  | Heldman DA | 2012 | accelerometer and gyroscope | gait, bradykinesia | 42:0 | Conf Proc IEEE Eng Med Biol Soc.302 2012;1956-9 |
|  | Wagner R | 2012 | tactile switch | gait | NA^1^ | Conf Proc IEEE Eng Med Biol Soc.2012;:280-3 |
|  | Kim JH | 2011 | gyroscope | bradykinesia | 40:14 | Med Biol Eng Comput. 2011;49:365-71 |
|  | Patel S | 2011 | accelerometer | motor fluctuation (bradykinesia) | 5:0 | Conf Proc IEEE Eng Med Biol Soc.2011;1552-5 |
|  | Zwartjes DG | 2010 | accelerometer and gyroscope | tremor, bradykinesia | 6:7 | IEEE Trans Biomed Eng. 2010;57 |
|  | Salarian A | 2007 | gyroscope | tremor, bradykinesia | 10:10  and 11:2^2^ | IEEE Trans Biomed Eng. 2007;54:313-22 |
| smartphone and wear-able device | Takač B | 2013 | depth sensor, smartphone (gyroscope, accelerome-ter, magnetometer) | freezing of gait | 12:0 | JMIR Mhealth Uhealth. 2013;1:e14 |

1) NA, not available

2) separate studies with different devices were conducted.
