# Supplementary material for: A Validation Study of a Smartphone-Based Finger Tapping Application for Quantitative Assessment of Bradykinesia in Parkinson’s Disease
Source: PLoS One. 2016 Jul 28;11(7):e0158852. doi: 10.1371/journal.pone.0158852 (PMC4965104; doi:10.1371/journal.pone.0158852)
Supplement: S2 Table — (DOCX) [file pone.0158852.s003.docx]

S2 Table. Results of linear regression between numbers of taps using two different methods of a mechanical tapper and smartphone tapper in idiopathic Parkinson’s disease and controls. Median values of three trials were used in the analyses.

|  | Types of measurement with a mechanical tapper | Average | | | | Better hand | | | | Worse hand | | | |
| --- | --- | --- | --- | --- | --- | --- | --- | --- | --- | --- | --- | --- | --- |
|  |  | Estimate | SE | R^2^ | p value | Estimate | SE | R^2^ | p value | Estimate | SE | R^2^ | p value |
| PD | MeT1P | 0.45 | 0.07 | 0.45 | <0.0001 | 0.49 | 0.07 | 0.49 | <0.0001 | 0.42 | 0.07 | 0.38 | <0.0001 |
|  | MeT2P | 1.14 | 0.18 | 0.41 | <0.0001 | 1.14 | 0.19 | 0.39 | <0.0001 | 1.08 | 0.20 | 0.33 | <0.0001 |
| control | MeT1P | 0.54 | 0.13 | 0.24 | 0.0001 | 0.58 | 0.14 | 0.23 | 0.0001 | 0.39 | 0.10 | 0.19 | 0.0004 |
|  | MeT2P | 1.67 | 0.19 | 0.57 | <0.0001 | 1.69 | 0.21 | 0.53 | <0.0001 | 1.52 | 0.19 | 0.52 | <0.0001 |

MeT1P, one-point tap measure of a mechanical tapper; MeT2P, two-points tap measure of a mechanical tapper.
