# Supplementary material for: A Validation Study of a Smartphone-Based Finger Tapping Application for Quantitative Assessment of Bradykinesia in Parkinson’s Disease
Source: PLoS One. 2016 Jul 28;11(7):e0158852. doi: 10.1371/journal.pone.0158852 (PMC4965104; doi:10.1371/journal.pone.0158852)
Supplement: S3 Table — (DOCX) [file pone.0158852.s004.docx]

**S3 Table. Results of linear regression between number of taps in three different timed tapping tests using a mechanical or smartphone tapper and limb’s item sum of motor scores of Unified Parkinson’s disease rating scale in idiopathic Parkinson’s disease.**

|  | limb’s item  sum | tapping tests | Right | | | | Left | | | |
| --- | --- | --- | --- | --- | --- | --- | --- | --- | --- | --- |
|  |  |  | Estimate | SE | R square | p value | Estimate | SE | R square | p value |
| R  I  G  H  T | motor UPDRS | MeT1P | -0.12 | 0.04 | 0.14 | **3.1.E-03** | -0.07 | 0.04 | 0.03 | 9.7.E-02 |
|  |  | MeT2P | -0.41 | 0.09 | 0.25 | **4.3.E-05** | -0.18 | 0.10 | 0.04 | 8.3.E-02 |
|  |  | SmT | -0.18 | 0.05 | 0.15 | **1.9.E-03** | -0.10 | 0.06 | 0.03 | 1.1.E-01 |
|  | bradykinesia | MeT1P | -0.12 | 0.03 | 0.27 | **2.2.E-05** | -0.08 | 0.03 | 0.14 | **2.3.E-03** |
|  |  | MeT2P | -0.33 | 0.06 | 0.33 | **1.7.E-06** | -0.22 | 0.07 | 0.14 | **2.8.E-03** |
|  |  | SmT | -0.17 | 0.04 | 0.30 | **6.3.E-06** | -0.12 | 0.04 | 0.13 | **3.3.E-03** |
|  | rigidity score | MeT1P | -0.02 | 0.01 | 0.02 | 1.7.E-01 | -0.01 | 0.01 | -0.01 | 5.1.E-01 |
|  |  | MeT2P | -0.08 | 0.03 | 0.10 | **1.1.E-02** | -0.03 | 0.03 | 0.00 | 3.9.E-01 |
|  |  | SmT | -0.01 | 0.02 | -0.01 | 4.1.E-01 | 0.00 | 0.02 | -0.02 | 7.9.E-01 |
|  | tremor score | MeT1P | 0.01 | 0.01 | 0.00 | 3.6.E-01 | 0.03 | 0.01 | 0.04 | 7.0.E-02 |
|  |  | MeT2P | 0.00 | 0.04 | -0.02 | 9.3.E-01 | 0.06 | 0.04 | 0.03 | 9.9.E-02 |
|  |  | SmT | 0.01 | 0.02 | -0.01 | 5.9.E-01 | 0.03 | 0.02 | 0.02 | 1.4.E-01 |
| L  E  F  T | motor UPDRS | MeT1P | -0.13 | 0.04 | 0.12 | **5.1.E-03** | -0.15 | 0.04 | 0.18 | **6.7.E-04** |
|  |  | MeT2P | -0.42 | 0.11 | 0.21 | **2.2.E-04** | -0.43 | 0.10 | 0.22 | **1.2.E-04** |
|  |  | SmT | -0.22 | 0.06 | 0.19 | **4.2.E-04** | -0.23 | 0.06 | 0.19 | **3.9.E-04** |
|  | bradykinesia | MeT1P | -0.12 | 0.03 | 0.17 | **8.6.E-04** | -0.12 | 0.03 | 0.19 | **4.0.E-04** |
|  |  | MeT2P | -0.33 | 0.09 | 0.20 | **3.4.E-04** | -0.33 | 0.08 | 0.20 | **2.5.E-04** |
|  |  | SmT | -0.21 | 0.05 | 0.26 | **3.2.E-05** | -0.17 | 0.05 | 0.18 | **6.8.E-04** |
|  | rigidity score | MeT1P | -0.02 | 0.01 | 0.03 | 1.1.E-01 | -0.02 | 0.01 | 0.06 | **3.5.E-02** |
|  |  | MeT2P | -0.09 | 0.03 | 0.14 | **2.4.E-03** | -0.06 | 0.03 | 0.06 | **3.3.E-02** |
|  |  | SmT | -0.03 | 0.02 | 0.03 | 1.2.E-01 | -0.04 | 0.02 | 0.07 | **2.6.E-02** |
|  | tremor score | MeT1P | 0.01 | 0.01 | 0.00 | 3.4.E-01 | 0.00 | 0.01 | -0.02 | 8.0.E-01 |
|  |  | MeT2P | -0.01 | 0.03 | -0.02 | 7.8.E-01 | -0.04 | 0.03 | 0.03 | 1.2.E-01 |
|  |  | SmT | 0.01 | 0.01 | -0.01 | 6.3.E-01 | -0.02 | 0.01 | 0.01 | 2.6.E-01 |

MeT1P, one-point tap measure of a mechanical tapper; MeT2P, two points tap measure of a mechanical tapper, SmT, smartphone tapper. P values less than 0.05 were marked in bold.
