# Supplementary material for: A Validation Study of a Smartphone-Based Finger Tapping Application for Quantitative Assessment of Bradykinesia in Parkinson’s Disease
Source: PLoS One. 2016 Jul 28;11(7):e0158852. doi: 10.1371/journal.pone.0158852 (PMC4965104; doi:10.1371/journal.pone.0158852)
Supplement: S4 Table — (DOCX) [file pone.0158852.s005.docx]

S4 Table. Differences in number of taps in the smartphone tapper test according to sex in normal controls.

|  | **ALL** | **men** | **women** | **p value*** |
| --- | --- | --- | --- | --- |
| Average | 54.54 (11.58) | 58.18 (9.28) | 52.21 (12.37) | 0.0126 |
| dominant hand | 59.17 (13.13) | 63.71 (10.68) | 56.26 (13.82) | 0.0060 |
| non-dominant hand | 49.75 (10.65) | 52.71 (8.28) | 47.85 (11.61) | 0.0255 |

Values are mean tapping numbers with standard deviation in parenthesis.
