# Supplementary material for: A Validation Study of a Smartphone-Based Finger Tapping Application for Quantitative Assessment of Bradykinesia in Parkinson’s Disease
Source: PLoS One. 2016 Jul 28;11(7):e0158852. doi: 10.1371/journal.pone.0158852 (PMC4965104; doi:10.1371/journal.pone.0158852)
Supplement: S5 Table — (DOCX) [file pone.0158852.s006.docx]

S5 Table. Changes in number of taps in the smartphone tapper test according to age in normal controls.

|  | **Estimate** | **SE** | **adjusted R squared** | **p values** |
| --- | --- | --- | --- | --- |
| Average | -0.54 | 0.06 | 0.48 | 0.0000 |
| dominant hand | -0.61 | 0.07 | 0.46 | 0.0000 |
| non-dominant hand | -0.46 | 0.06 | 0.41 | 0.0000 |
