# Supplementary material for: A Validation Study of a Smartphone-Based Finger Tapping Application for Quantitative Assessment of Bradykinesia in Parkinson’s Disease
Source: PLoS One. 2016 Jul 28;11(7):e0158852. doi: 10.1371/journal.pone.0158852 (PMC4965104; doi:10.1371/journal.pone.0158852)
Supplement: S6 Table — (DOCX) [file pone.0158852.s007.docx]

S6 Table. Factors affecting tap number in smartphone tapper test in patients with idiopathic Parkinson’s disease.

|  | parameters | Average | | | Better hand | | | Worse hand | | |
| --- | --- | --- | --- | --- | --- | --- | --- | --- | --- | --- |
|  |  | Esti-mate | SE | p value | Esti-mate | SE | p value | Esti-mate | SE | p value |
| Uni-variate analy-ses | age | -0.10 | 0.12 | 0.4210 | -0.11 | 0.13 | 0.4150 | -0.03 | 0.12 | 0.7890 |
|  | gender | 3.12 | 2.14 | 0.1510 | 3.07 | 2.35 | 0.1960 | 2.45 | 2.23 | 0.2780 |
|  | duration* | -0.05 | 0.02 | 0.0395 | -0.07 | 0.02 | 0.0048 | -0.03 | 0.02 | 0.2710 |
|  | H&Y | -7.75 | 1.82 | 0.0001 | -7.66 | 2.05 | 0.0004 | -7.06 | 1.95 | 0.0006 |
|  | mUPDRS | -0.39 | 0.09 | 0.0000 | -0.44 | 0.09 | 0.0000 | -0.30 | 0.10 | 0.0031 |
| Multi-variate analy-ses | mUPDRS | -0.38 | 0.10 | 0.0004 | -0.38 | 0.11 | 0.0009 | -0.32 | 0.11 | 0.0060 |
|  | duration* | 0.00 | 0.02 | 0.8591 | -0.03 | 0.03 | 0.2995 | 0.01 | 0.03 | 0.6954 |

mUPDRS, motor scores of Unified Parkinson’s disease rating scale. *, duration of Parkinson’s disease
